# Fast and reliable quantitative measures of white matter development with magnetic resonance fingerprinting

**DOI:** 10.1162/imag_a_00470

**Published:** 2025-02-18

**Authors:** Maya Yablonski, Zihan Zhou, Xiaozhi Cao, Sophie Schauman, Congyu Liao, Kawin Setsompop, Jason D. Yeatman

**Affiliations:** Division of Developmental-Behavioral Pediatrics, Department of Pediatrics, Stanford School of Medicine, Palo Alto, CA, United States; Graduate School of Education, Stanford University, Stanford, CA, United States; Department of Radiology, Stanford University, Stanford, CA, United States; Department of Electrical Engineering, Stanford University, Stanford, CA, United States; Department of Psychology, Stanford University, Stanford, CA, United States

**Keywords:** magnetic resonance fingerprinting, quantitative MRI, white matter development, T1 mapping, qT1

## Abstract

Developmental cognitive neuroscience aims to shed light on evolving relationshipsbetween brain structure and cognitive development. To this end, quantitativemethods that reliably measure individual differences in brain tissue propertiesare fundamental. Standard qualitative MRI sequences are influenced by scanparameters and hardware-related biases, and also lack physical units, making theanalysis of individual differences problematic. In contrast, quantitative MRIcan measure physical properties of the tissue but with the cost of long scandurations and sensitivity to motion. This poses a critical limitation forstudying young children. Here, we examine the reliability of an efficientquantitative multiparameter mapping method—magnetic resonancefingerprinting (MRF)—in children scanned longitudinally. We focus on T1values in white matter, since quantitative T1 values are known to primarilyreflect myelin content, a key factor in brain development. Forty-nine childrenaged 8–13 years (mean 10.3 years ± 1.4) completed 2 scanningsessions 2–4 months apart. In each session, two 2-min 3D-MRF scans at 1mm isotropic resolution were collected to evaluate the effect of scan durationon image quality and scan–rescan reliability. A separate calibration scanwas used to measure B0 inhomogeneity and correct for bias. We examined theimpact of scan time and B0 inhomogeneity correction on scan–rescanreliability of values in white matter, by comparing single 2-min and combinedtwo 2-min scans, with and without B0 correction. Whole-brain voxel-basedreliability analysis showed that combining two 2-min MRF scans improvedreliability (Pearson’s r = 0.87) compared with a single 2-min scan(r = 0.84), while B0 correction had no effect on reliability in whitematter (r = 0.86 and 0.83 4- vs. 2-min). Using diffusion tractography, wesegmented major white matter fiber tracts and examined the profiles ofMRF-derived T1 values along each tract. We found that T1 values from MRF showedsimilar or greater reliability compared with diffusion parameters. Lastly, wefound that R1 (1/T1) values in multiple white matter tracts were significantlycorrelated with age. In sum, MRF-derived T1 values were highly reliable in alongitudinal sample of children and replicated known age effects. Reliability inwhite matter was improved by longer scan duration but was not affected by B0correction, making it a quick and straightforward scan to collect. We proposethat MRF provides a promising avenue for acquiring quantitative brain metrics inchildren and patient populations where scan time and motion are of particularconcern.

## 1 Introduction

Developmental cognitive neuroscience aims to shed light on evolving relationshipsbetween brain structure and cognitive development. To this end, quantitative methodsthat reliably measure individual differences in the structure of brain tissue arefundamental. Commonly used qualitative MRI sequences, for example, T1-weighted andT2-weighted contrasts, are useful for delineating anatomy and detecting pathologies.However, these qualitative measures do not provide quantitative metrics thatreliably index individual differences in tissue properties (Lerch et al., 2017). QualitativeMRI measures are influenced by scan parameters and hardware-related biases (forreview, seeCheng et al., 2012)and also lack physical units that can accurately quantify differences amongparticipants. Images also may appear dramatically different due to specific scannerand protocol settings, making it hard to repeat and compare across studies, andwithin the same individual over time.

In contrast, quantitative MRI (qMRI) techniques aim to quantify specific physicalproperties of the tissue that shed light on cellular structure. Specifically,quantitative longitudinal relaxation mapping (T1) has physical units (milliseconds)that are reliable across different hardware setups (Mezer et al., 2013) and have been reported to beprimarily sensitive to myelin content in the white matter (Lutti et al., 2014;Stüber et al., 2014;Warntjes et al., 2017). Thisspecificity is crucial to uncover the neurobiological mechanisms of braindevelopment, degeneration, and disease. For example, changes in T1 values have beendescribed in multiple sclerosis (Harperet al., 2023;Stevenson etal., 2000), reflecting alterations in axon and myelin composition.Similarly, quantitative T1 has been used to study aging (Eminian et al., 2018;Filo et al., 2019), anddegenerative and inflammatory processes in clinical conditions (Gozdas et al., 2021;Menke et al., 2009;Moallemian et al., 2023), as wellas lifespan maturation and degeneration of white matter (Yeatman, Wandell, et al., 2014).Combining quantitative T1 with diffusion measurements can provide complementaryinformation and support inferences about biological processes. For example, T1 candifferentiate between white matter tracts that have similar diffusion values (Schurr et al., 2019;Yeatman, Weiner, et al., 2014),while diffusion metrics seem to be more sensitive to learning induced plasticity(Huber et al., 2021).

Although quantitative T1 mapping can provide insights into neurobiologicalmechanisms, it is not commonly incorporated into studies of child development.Existing T1 protocols typically require long acquisition times, which vary from 10up to 25 min, depending on the protocol type and resolution (e.g.,Callaghan et al., 2014;Deoni et al., 2005;Gracien et al., 2017;Helms et al., 2008;Kecskemeti et al., 2016;Leutritz et al., 2020;Marques & Gruetter, 2013;Mezer et al., 2013;O’Muircheartaigh et al.,2019;Sanchez Panchuelo etal., 2021). This limits the feasibility for children and clinicalpopulations that have difficulties staying in the scanner for long periods of time.Moreover, children tend to move while in the scanner (Barkovich et al., 2019;Greene et al., 2018), which is a major challenge for manyquantitative measures that require multiple images to be collected in sequence. Forall these reasons, loss of data is a major barrier to large-scale quantitative MRIstudies in children. When scans are required for medical reasons, it is a commonpractice to sedate children in order to get good data quality, though sedation alsohas health implications (Artunduaga etal., 2021;Jaimes et al.,2018;Wilder et al.,2009) and is, therefore, rarely used in research. This makes thedevelopment of reliable sequences with short scan times of particular importance forpediatric research and practice.

Magnetic resonance fingerprinting (MRF) is an efficient quantitative MRI techniquethat has the potential to address these limitations by providing a rapid andhigh-resolution data acquisition, particularly with recent advancements that havefurther improved its acquisition efficiency (Cao et al., 2019,^2022^;Liaoet al., 2024;Ma et al.,2013,^2018^). MRF isdesigned to simultaneously quantify different tissue properties in a single fastacquisition. With recent improvements, it can achieve whole-brain 1-mm isotropicmapping in 2 min, which paves the way for using quantitative MRI across a broadrange of research and, eventually, in clinical practice. Several studies haveassessed the reliability and reproducibility of MRF (Buonincontri et al., 2019;Fujita et al., 2023;Gracien et al., 2020;Körzdörfer et al., 2019;Wicaksono et al., 2023), andothers reported excellent agreement between MRF values compared with gold standardquantitative sequences (Jiang et al.,2017;Liao et al.,2017;Ma et al.,2013;Statton et al.,2022;Zhou et al.,2023), but these analyses have been done in phantoms and healthy humanadults. Despite the accumulating evidence validating MRF as a stable technique, theuse of MRF in pediatric studies has been scarce (Chen et al., 2019;Kim et al., 2023;Liao et al., 2024). To date, the reliability and qualityof MRF-based measurements in pediatric and clinical populations, where data qualityis a major challenge, have never been evaluated. The aim of the current study is toaddress this gap by providing a thorough and rigorous assessment of MRFscan–rescan reliability in a sample of children. This is an essential firststep for adopting MRF in developmental studies, since the reliability of ameasurement is the upper limit for detecting any correlations with othermeasurements (e.g., brain–behavior correlations) and how they evolve overtime.

To this end, in the current study we evaluate the scan–rescan reliability ofMRF-estimated T1-maps in a group of children scanned twice, several months apart. Weshow that MRF-derived T1 values are (1) highly reliable, (2) precisely captureindividual differences in tissue properties, and (3) replicate known age effects inwhite matter tracts. In addition, we examine different reconstruction pipelines andevaluate the influence of different pipeline choices on scan–rescanreliability. We conclude with recommendations for incorporating MRF acquisitions instudies of brain development in health and disease.

## 2 Methods

### 2.1 Participants

In total, 49 children aged 8–13 years (mean 10.3 years ± 1.4; 29/20female/male) completed 2 scanning sessions 2–4 months apart at theStanford University Center for Neurobiological Imaging, as part of an ongoinglongitudinal study. All children provided informed assent to participate in thestudy and their parents provided written consent. The study protocol wasapproved by the Stanford University School of Medicine Institutional ReviewBoard. Before the first scan, children were acclimated to the MRI environmentwith a mock MRI scanner. All data were acquired on a 3T GE Discovery MR750 UHP(GE Healthcare, Milwaukee, WI, USA), equipped with a Nova 32-channel headcoil.

### 2.2 Data acquisition

#### 2.2.1 MRF acquisition

In each session, we collected two 3D-MRFs and a separate“PhysiCal” calibration scan. MRF data were acquired using tinygolden-angle shuffling MRF with optimized spiral-projection trajectories asproposed inCao et al.(2022). In the MRF sequence, an adiabatic inversion-preparedpulse is used with an inversion time of 15 ms, followed by acquisitiongroups. Each acquisition group contains 500 TRs with varying flip angles. Aresting time of 1.2 s follows each acquisition group to allow for signalrecovery before the next acquisition group. Different acquisition groups usedifferent complementary spiral k-space trajectories. A total of 16acquisition groups were used in each MRF sequence to obtain 1-mm whole-brainquantitative (2-min scan time). The field of view (FOV) was 220 × 220× 220 mm^3^, with reconstruction matrix of 220 × 220× 200. While the two MRF scans used the same parameters, theyincluded complementary k-space acquisition trajectories to allow them to becombined synergistically to produce a 4-min scan with reduced k-tundersampling. We chose to combine two 2-min acquisition rather than asingle 4-min acquisition due to evidence that shorter sequences with morebreaks decrease motion and improve scan quality in children (Meissner et al., 2020). Inaddition, a unified, rapid calibration sequence called Physics Calibration(PhysiCal) (Iyer et al.,2020) was used to measure B0 inhomogeneity within the same FOV,with resolution of 2 × 2 × 2 mm^3^in 25 s.

#### 2.2.2 Diffusion-weighted acquisition

We collected multi-shell dMRI data with 180 diffusion-weighted volumes,distributed across 3 shells with the following gradient scheme: 30directions with b = 1000 s/mm^2^, 60 directions with b= 2000 s/mm^2^, and 90 directions with b = 3000s/mm^2^, as well as 14 reference volumes without diffusionweighting (b = 0 s/mm^2^). We used a repetition time (TR) of3335 ms with echo time (TE) of 87 ms to obtain spatial resolution of 1.5mm^3^isotropic voxels in 84 axial slices. This was achievedusing a hyperband acceleration with a slice acceleration factor of 4. Thescan duration totaled 11 min and was broken down to two 5.5-min scans toallow children a short break. An additional scan of sixnon-diffusion-weighted volumes with a reversed phase encoding direction andthe same parameters was also acquired to correct for echo-planar imaging(EPI) distortions.

#### 2.2.3 Anatomical acquisition

A high-resolution T1-weighted (T1w) anatomical scan was acquired usingGE’s BRAVO sequence, which is a fast spoiled gradient echo (SPGR)with a spatial resolution of 0.9 mm^3^isotropic voxels and thefollowing parameters: inversion time (TI) of 450 ms, flip angle of 12degrees, receiver bandwidth of 41.67 kHz, field of view 23 cm, and a matrixof 256 × 256. The scan duration was 4:47 min.

### 2.3 Data processing

#### 2.3.1 MRF image reconstruction

##### 2.3.1.1 B0 field inhomogeneity estimation

From the PhysiCal data, highly under-sampled multi-echo images werereconstructed with parallel imaging and compressed sensing methods asdescribed inIyer et al.(2020). Subsequently, a robust multi-echo general linearmodeling robustly recovered an artifact-free B0 map (Corbin et al.,2019).

##### 2.3.1.2 Subspace reconstruction without B0 inhomogeneity correction

The reconstruction used a spatiotemporal subspace modeling with locallylow rank (LLR) constraint based on previous literature (Cao et al., 2022). TheMRF dictionary was pre-calculated using the extended phase graph method(Weigel, 2015).The T1 dictionary entries covered the range of 20 to 3000 ms, with astep size of 20 ms. The dictionary also covered higher values between3000 and 5000 ms (corresponding to CSF), with a coarse step size of 200ms. A singular value decomposition (McGivney et al., 2014) was applied on thepre-calculated dictionary to get the first five temporal principalcomponents, termed as subspace basis Φ. The coefficient mapsCof the bases can be reconstructed by this formula:

$$\underset{c}{\min}||PFS\phi c-y{||}_{2}^{2}-\lambda R_{r}\left(c\right),$$

wherePis the under-sampling pattern,Fis the nonuniform Fourier transform (NUFFT),Sis the coil sensitivity map,yis the acquired k-space data,$R_{r}\left(c\right)$is LLR term, andλis the regularization.

##### 2.3.1.3 Subspace reconstruction with B0 inhomogeneity correction

With the presence of B0 field inhomogeneity, the acquired signal becomes$e^{-it\omega_{m}}y$and the equation becomes

$$\underset{c}{\min}||PFS\phi c-e^{-it\omega_{m}}y{||}_{2}^{2}-\lambda R_{r}\left(c_{m}\right),$$

where$e^{-it\omega_{m}}y$is the conjugate phase demodulation with acquisitiontime*t*accumulated during the acquisition trajectory atan off-resonance frequency$\omega_{m}$.In this study, we incorporated a time-segmented B0 correction method(Irarrazabal et al.,1996;Noll etal., 1991) to correct the B0-inhomogeneity induced imageblurring.

##### 2.3.1.4 Template matching

Subspace-based compression was also applied to the pre-calculateddictionary, which can significantly reduce the computational load andmemory requirements. Then, a cross-correlation template matching wasapplied to the reconstructed coefficient maps using the compresseddictionary to obtain quantitative T1 values, as described inMa et al. (2013).

##### 2.3.1.5 MRF reconstruction pipelines

In order to assess the scan time effects on image quality, data werereconstructed in four ways: (1) using a single 2-min MRF data. (2)Combining two 2-min MRF scans (4-min). (3) Using a single 2-min MRF incombination with B0 correction. (4) Combining two 2-min MRF scans withB0 correction. Importantly, to account for any motion between the two2-min MRF scans, we applied the following procedure to coregister thesecond scan to the first one, before reconstructing the combined 4-mindata: We first reconstructed the two 2-min MRFs separately and thenestimated the rigid transformation motion parameters between them usingAFNI 3dvolreg (Cox,1996;Cox& Hyde, 1997). We then used these parameters to updatethe second MRF acquisition’s trajectory and data in k-space.Finally, we combined the k-space data and trajectories of the first andsecond 2-min acquisitions to reconstruct the combined 4-min data. Thisapproach ensured that the combined 4-min data are in the exact samespace as the first 2-min data to which it was compared in our subsequentanalyses. This registration procedure improved the quality of the 4-minreconstruction and reduced blurring (as observed qualitatively).

These computations were performed on a Linux server using MATLAB R2022a(The MathWorks, Natick, MA, USA) and Python scripts (seehttps://github.com/SophieSchau/MRF_demo_ISMRM2022/), with thefollowing specifications: Ubuntu 20.04 with 104 Core Intel Xeon Gold2.20G CPUs, an Nvidia RTX A6000, and 503GB RAM. Reconstruction of each2-min acquisition took about 40 min with B0 correction and 10-minwithout B0 correction. The 4-min data reconstructions took 57 min withB0 correction and 16 min without B0 correction.

#### 2.3.2 dMRI preprocessing

Diffusion data were pre-processed using the default pipeline in QSIprep0.19.1 (Cieslak et al.,2021). The T1w image was corrected for intensity non-uniformityusing*N4BiasFieldCorrection*(Tustison et al., 2010; ANTs 2.4.3) and reorientedinto AC-PC alignment. This image was used as an anatomical referencethroughout the workflow. The diffusion data were denoised with MP-PCAdenoising as implemented in MRtrix3’s*dwidenoise*(Tournier et al.,2019;Veraart etal., 2016) with a 5-voxel window. After denoising, the meanintensity of the diffusion-weighted series was adjusted so that the meanintensity of the b = 0 images matched across each separate DWIscanning sequence. B1 field inhomogeneity was corrected using*dwibiascorrect*from MRtrix3 with the N4 algorithm(Tustison et al.,2010). FSL’s Eddy (version 6.0.5.1:57b01774) was used forhead motion correction and Eddy current correction (Andersson & Sotiropoulos,2016). Eddy was configured with a q-space smoothing factor of 10,a total of 5 iterations, and 1000 voxels used to estimate hyperparameters. Alinear first level model and a linear second level model were used tocharacterize Eddy current-related spatial distortion. q-Space coordinateswere forcefully assigned to shells. Eddy’s outlier replacement wasrun (Andersson et al.,2016). Data were grouped by slice, only including values fromslices determined to contain at least 250 intracerebral voxels. Groupsdeviating by more than 4 standard deviations from the prediction had theirdata replaced with imputed values. b = 0 reference images withreversed phase encoding directions were used along with an equal number of b= 0 images extracted from the primary diffusion scans. Thesusceptibility-induced off-resonance field was estimated using a methodsimilar to that described inAndersson et al. (2003). The field maps were ultimatelyincorporated into the Eddy current and head motion correction interpolation.The final interpolation was performed using the jac method. Finally, the twodiffusion-weighted series were concatenated and resampled to the anatomicalAC-PC space with 1.5 mm isotropic voxels.

#### 2.3.3 Fiber tractography

Voxel-level diffusion modeling and whole-brain tractography were carried outusing QSIprep’s MRtrix3 (Tournier et al., 2019) reconstruction pipeline. Within eachvoxel, diffusion was modeled with constrained spherical deconvolution (CSD)using the*dhollander*algorithm for estimating the fiberresponse function (Dhollanderet al., 2016,^2019^). Whole-brain tractography was carried out using the iFOD2algorithm with the multi-shell multi-tissue method (msmt-csd;Tournier et al., 2004,^2008^). One millionstreamlines were generated and their length was limited to the range50–250 mm, resulting in a whole-brain tractogram in eachsubject’s native space for each timepoint.

pyAFQ (Kruper et al.,2021;Yeatman,Dougherty, Myall, et al., 2012) was then applied to thewhole-brain tractograms to (i) segment white matter tracts of interest and(ii) calculate tract profiles of variation in diffusion metrics along thetrajectory of each tract. A standard cleaning procedure was used to removespurious streamlines that do not follow the trajectory of the desired tract.Specifically, we removed streamlines that deviated from the tract core bymore than 3 standard deviations, or that were longer than the mean tractlength by more than 4 standard deviations. This process was repeatediteratively five times.

Diffusion metrics, namely, fractional anisotropy (FA) and mean diffusivity(MD), were calculated by pyAFQ based on the diffusion kurtosis imaging model(DKI;Jensen et al.,2005) as implemented in DiPy (Garyfallidis et al., 2014;Henriques et al., 2021).These metrics were then sampled onto 100 equidistant nodes along each tract,such that for each metric the value at the node represents the weightedaverage across the streamlines that comprise the tract. Here we limit ouranalyses to the middle 80 nodes of each tract, as nodes closer to the tractterminations typically suffer from partial volume as streamlines enter graymatter. To evaluate reliability across different regions of the brain, wesegmented both inter- and intra-hemispheric tracts. First, we segmented theseven sub-bundles of the corpus callosum (CC): Orbital, Superior Frontal,Motor, Superior Parietal, Posterior Parietal, Temporal, and Occipital (Dougherty et al., 2007).Then, we also segmented seven intra-hemispheric tracts that have beenfrequently studied in developmental cognitive neuroscience: the direct andposterior branches of arcuate fasciculus (ARC, pARC), the superiorlongitudinal fasciculus (SLF), the inferior fronto-occipital fasciculus(IFOF), the inferior longitudinal fasciculus (ILF), the uncinate fasciculus(UF), and the corticospinal tract (CST). Each tract was segmentedbilaterally (seeFig.1).

**Fig. 1.**
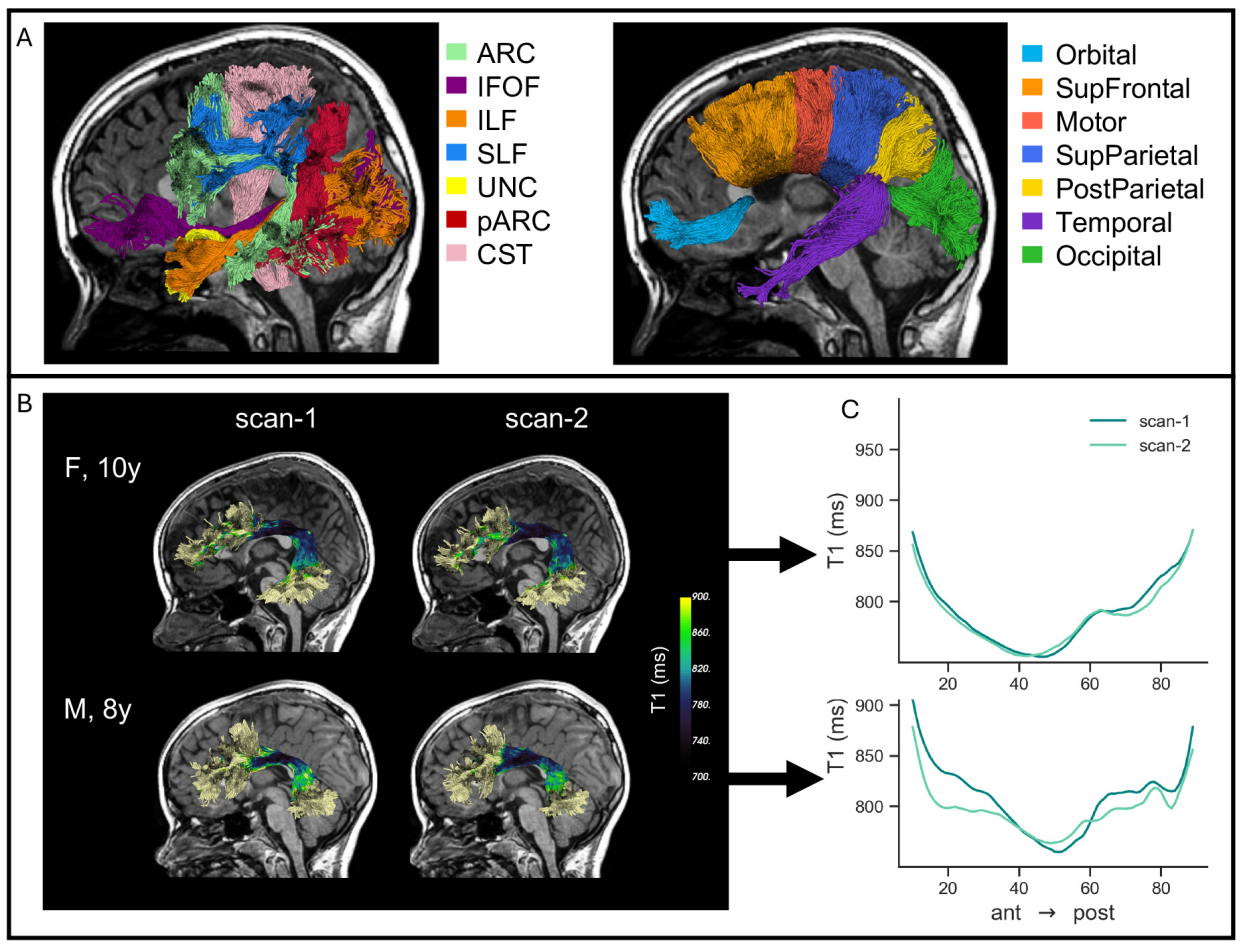
Calculating T1 profiles along the trajectory of major white mattertracts. (A) Intra-hemispheric tracts (left) and callosal sub-bundles(right) of a representative subject, overlaid on their anatomicalT1w image. (B) Left arcuate fasciculus (ARC) of two example subjectsacross scans at two timepoints. The tract streamlines are colorcoded by their corresponding T1 values. Tract shape is variablebetween subjects yet highly consistent within the same subject overtime. (C) T1 profiles along the trajectory of the left ARC. Nodesare ordered from anterior to posterior position.

### 2.4 Reliability analysis

We took three approaches to evaluate scan–rescan reliability in whitematter: (1) voxel-wise analysis across the entire white matter, (2) ROI-basedanalysis of the corpus callosum (CC), and (3) tractometry-based comparisons inmultiple white matter tracts (Fig.1). In each of these three analyses, we compared values obtained fromthe first scanning session available for each child (“scan-1”)with the values obtained in their second scan, which took place 2–4months apart (“scan-2”). When comparing the 2-min reconstructionpipelines, we always compared the first 2-min acquisition obtained in eachscanning session. This provides a lower limit on scan–rescan reliabilityas there might be some developmental changes over this time scale. For each ofthese approaches, we calculate several measures of scan–rescanreliability: Pearson’s correlation coefficient (r), the coefficient ofdetermination (R^2^), and the coefficient of variation (CV) between thetwo scans. The coefficient of variation is defined as the ratio between thestandard deviation of two measurements, and their mean. Lower CV is, therefore,considered better as it indicates less variation in the measurement overrepeating scans. We visualize the distribution of the differences in T1 valuesbetween the two scans using Bland–Altman plots. We repeat thesecalculations for T1 values estimated using each of the four pipelines to explorewhether different reconstruction approaches impact reliability.

#### 2.4.1 Voxel-wise and ROI-based comparison

Accurate registration of the two sessions is an essential step for assessingscan–rescan reliability. We took several steps to achievehigh-quality registration (seeFig. 2): First, we registered and resampled the two scans into a“half-way” space using the ANTS unbiased pairwise registrationtool (Tustison et al.,2021), to maintain inverse consistency, instead of resampling thesource (the second scan) to the estimated target location (the first scan)(Reuter & Fischl,2011;Reuter et al.,2010). Second, instead of registering MRF-generated T1 maps, weregistered the reconstructed coefficient maps before template matching,which reduces the partial volume effects and thus improves the accuracy ofT1 fitting (Q. Chen et al.,2023). The registration was calculated for the 4-minreconstruction pipeline and then applied to all pipelines. Then, to obtainaccurate white matter masks in the “half-way” space, the two“half-way” space 4-min coefficient maps were averaged togenerate a higher SNR image (equivalent to an 8-min scan), followed bytemplate matching and synthetic T1w-MPRAGE generation. This high-qualityimage was generated using Bloch simulations (Wang et al., 2014) and used as input for theFreesurfer segmentation pipeline using the SynthSeg method (Billot et al., 2023), toobtain the white matter mask and corpus callosum (CC) ROIs (seeFig. 2). CC ROIs werevisually inspected and manually edited to exclude corticospinal fluid (CSF)and fornix voxels.

**Fig. 2.**
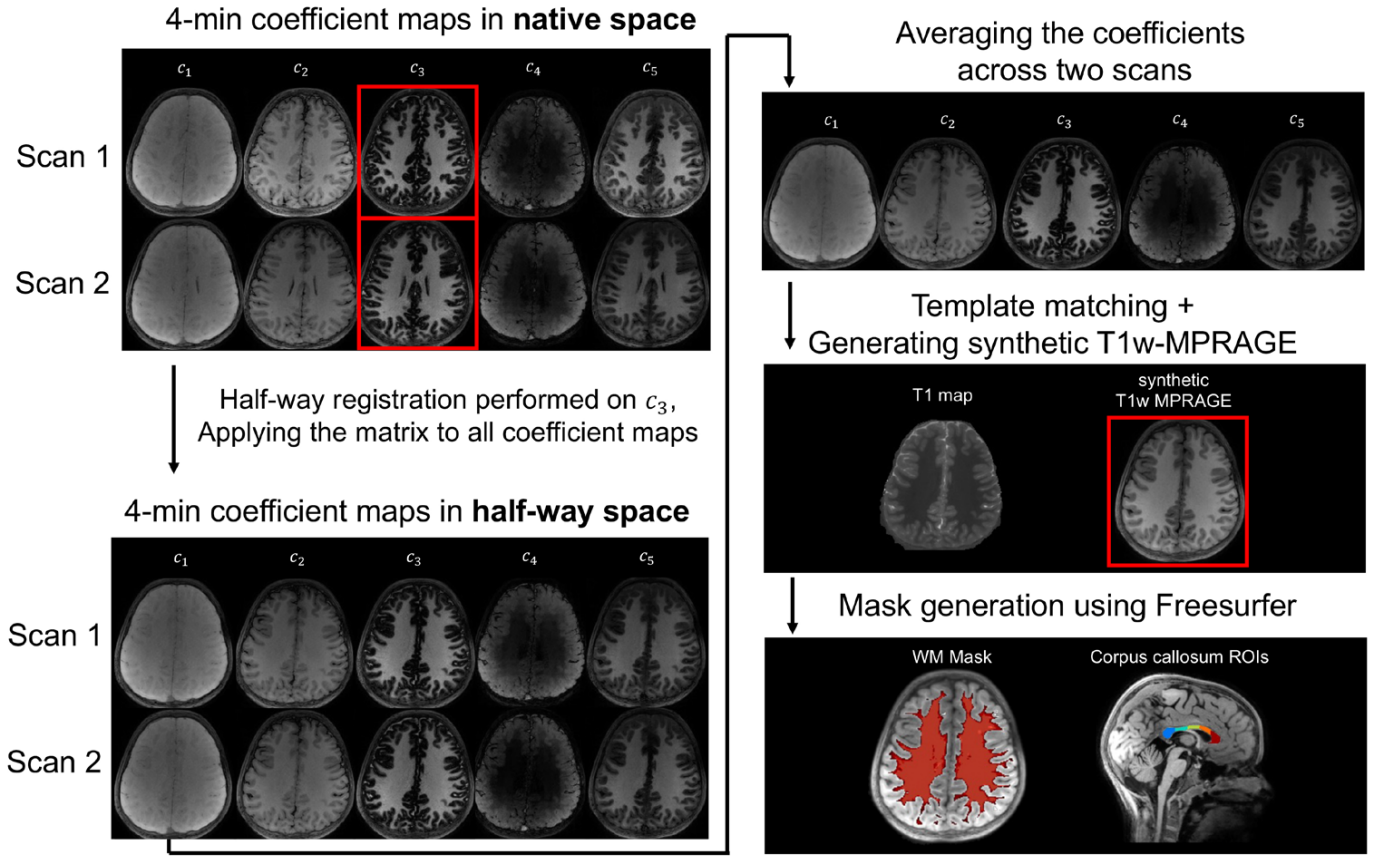
Workflow for the half-way registration process. Initially, 4-mincoefficient maps from two scans are presented in native space, wherethe*c*_3_coefficient (highlighted in a redbox) was chosen as the registration input due to its high contrast.Then, we applied the calculated transform matrices from each scan tothe half-way space to all five coefficient maps. We then averagedthe registered coefficient maps from both scans to increase SNR,before running the template matching to generate syntheticT1-weighted MPRAGE images. Lastly, masks were created usingFreesurfer software for white matter and corpus callosum regions ofinterest.

The scan–rescan reliability was first assessed voxel-wise within theentire white matter mask. Pearson’s correlation coefficient wascalculated among voxels of the two scan sessions for each participant.Coefficient of determination (R^2^) was further calculated with thefitting function y = x to assess the data consistency. We ran alinear mixed effect model using the lme4 package in R (Bates et al., 2015) toevaluate the contribution of scan duration and B0 correction toscan–rescan reliability. The model included age as a fixed effect aswell as a random intercept for each subject to account for individualvariability (full formula:

Pearson’s r~duration*B0 correction+age+(1|participant)).

For the ROI-based analysis, we focused on five corpus callosum (CC) regionsas the CC is an area with dense, highly myelinated axons (Aboitiz et al., 1992). Theaverage T1 value in each ROI was calculated per subject and compared acrossthe two scanning sessions. Again, we used Pearson’s correlation,coefficient of determination (R^2^), and coefficient of variation(CV) across all subjects as measures of scan–rescan reliability.While the main focus of the current study was white matter, we also analyzedseveral subcortical gray matter regions obtained with Freesurfer’spipeline to provide an estimate of the T1 values and reliability in graymatter.

#### 2.4.2 Tractography-based comparison

In order to evaluate T1 values along the tracts, we first had to register theMRF data to the native diffusion space. To achieve this, synthetic diffusionb = 0 images were generated based on the T1 and T2 maps from the4-min MRF scan. These synthetic images were registered to the diffusion b= 0 images using Advanced Normalization Tools (ANTs) (Avants et al., 2009;Klein et al., 2009). Arigid linear transformation was chosen with optimizing a mutual information(MI) similarity metric. The transformation was subsequently applied to theMRF coefficient maps, followed by template matching to get T1 values mappedinto the diffusion native space. T1 values obtained from each of the 4different pipelines were then sampled onto 100 equidistant nodes along eachtract using pyAFQ.

We evaluated scan–rescan reliability by comparing the mean T1 valuesof each tract across the two scans of each participant. We calculatedPearson’s correlation coefficient, coefficient of determination(R^2^), and coefficient of variation (CV) between the twoscans, when T1 values were estimated using each of the four pipelines.

### 2.5 Age effects

To examine whether MRF-derived values are able to replicate known developmentaleffects (Yeatman, Wandell, et al.,2014), we calculate Pearson’s correlation between mean R1values in each tract and participants’ age at the time of each scan. Forthis analysis we used R1, the inverse of T1 (1/T1), as our metric, since R1linearizes the T1 scale and has been more extensively studied in relation tobrain development (Travis et al.,2019;Yeatman, Wandell,et al., 2014). This was repeated for the four pipelines to testwhether different processing choices affect the ability to detect expected ageeffects.

## 3 Results

### 3.1 Qualitative evaluation of reconstructed maps

**Fig. 3.**
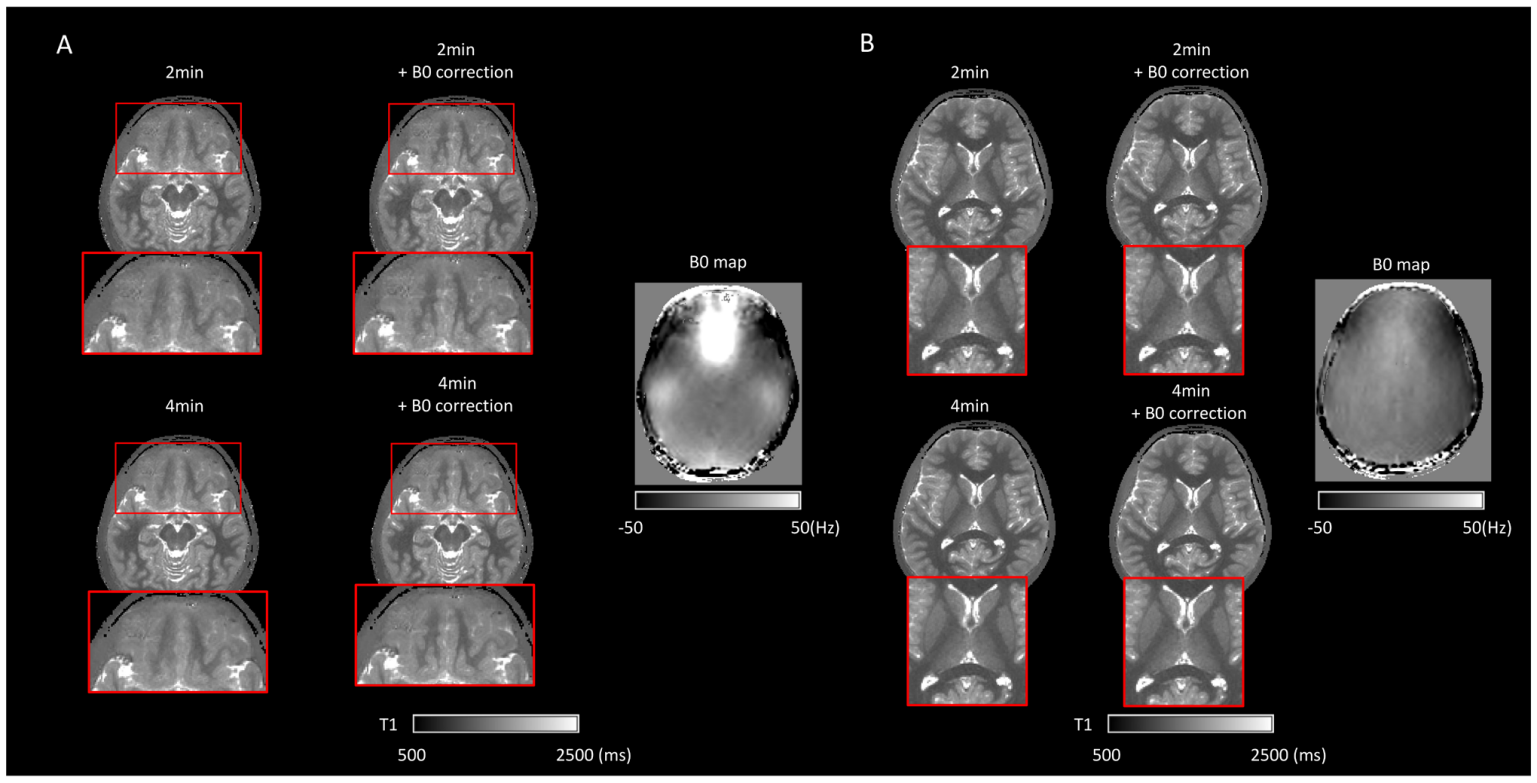
Example axial slices of reconstructed T1 maps using the four pipelines.(A) A slice passing through the sinuses, a region with high B0inhomogeneity (as shown in the corresponding B0 map on the right).Applying B0 correction significantly sharpens the image in theinhomogeneous area. (B) A more superior slice where B0 is morehomogeneous. Here, B0 correction does not show significant improvementto image quality in white matter. In both slices, maps generated from4-min data are less noisy than those of 2-min data.

Figure 3shows two typicalslices of reconstructed T1 maps from the four different pipelines. Visualinspection revealed that all the reconstructed 1 mm resolution images showedgood contrast between gray matter, white matter, and CSF. Compared with the2-min acquisition, the 4-min acquisition resulted in less noisy maps,irrespective of B0 correction (first row ofFig. 3A). B0 correction significantly reducedimage blurring around the sinuses, which are air-filled spaces in the skull thatinduce high B0 inhomogeneity (Fig.3A).

### 3.2 Voxel-wise comparison

**Fig. 4.**
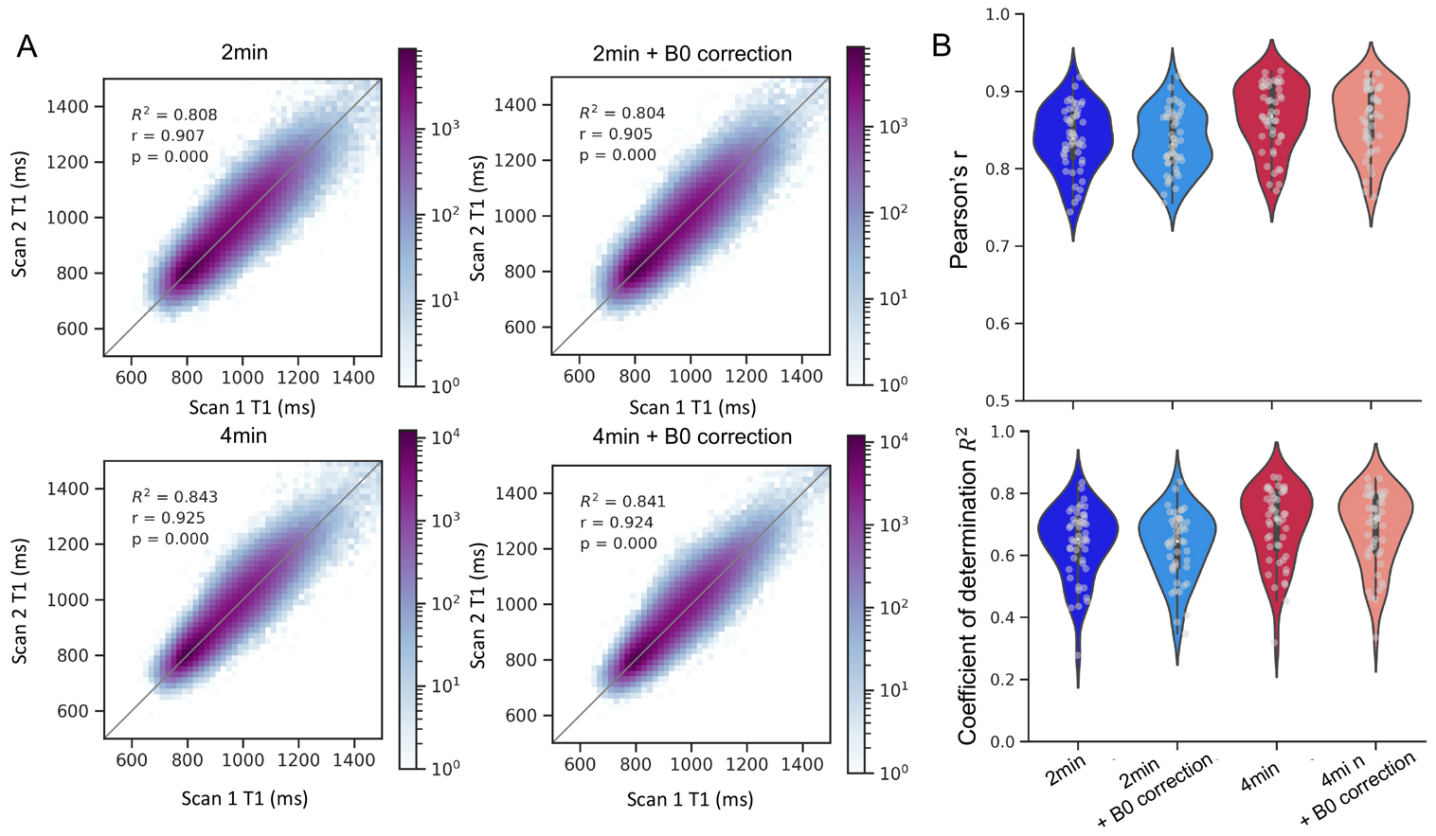
Voxel-wise scan–rescan reliability. (A) 2D Histograms show T1values in white matter voxels in the two scans of a single subject, forthe four reconstruction pipelines. Color denotes voxel count inlogarithmic scale. Gray lines denote the equality line. (B)Scan–rescan reliability across all subjects (N = 48),calculated as Pearson’s correlation (top panel) or coefficient ofdetermination (bottom panel).

Figure 4showsscan–rescan reliability across all white matter voxels in a singlesubject (panel A) and the distribution of correlation coefficients across allparticipants in the sample (panel B; one subject was removed from the analysisdue to severe blurring caused by motion). Individual participants showed highPearson’s correlation for the 2-min data (r = 0.838 ±0.041, range [0.744–0.918]) and coefficient of determination(R^2^= 0.638 ± 0.114, range [0.278–0.835]),indicating that individual variation in T1 values across voxels is highlyreliable across scans. As expected, reliability was higher for 4-min data,suggesting that longer scan duration improved SNR (r = 0.866 ±0.043, range [0.772–0.927]; R^2^= 0.693 ± 0.121,range [0.319–0.853]). B0 correction did not improve reliability comparedwith data without this correction (2-min: r = 0.838 vs. r = 0.834,R^2^= 0.638 vs. 0.629 for without vs. with B0 correction;4-min: r = 0.866 vs. 0.863, R^2^: 0.693 vs. 0.683 for withoutvs. with B0 correction). A linear mixed effect model confirmed that reliabilitywas higher for 4-min data compared with the 2-min data (β = 0.028,t = 12.85, p < 1e-16), but was not affected by the B0 correction(β = -0.004, t = -1.705, p = 0.09). The interactionbetween duration and B0 correction was not significant (β =0.0002, t = 0.065, p = 0.948) and neither was the effect of age(β = -0.006, t = -1.584, p = 0.12). Lastly, toexamine reliability in the absence of developmental changes, we also comparedthe values of the two 2-min scans that were collected at the same scanningsession. As expected, scan–rescan reliability was higher within sessioncompared with the 2-min data across sessions (r = 0.841 ± 0.031,range [0.696–0.880]; R^2^= 0.673 ± 0.065, range[0.365–0.755]).

### 3.3 ROI-based comparison

**Fig. 5.**
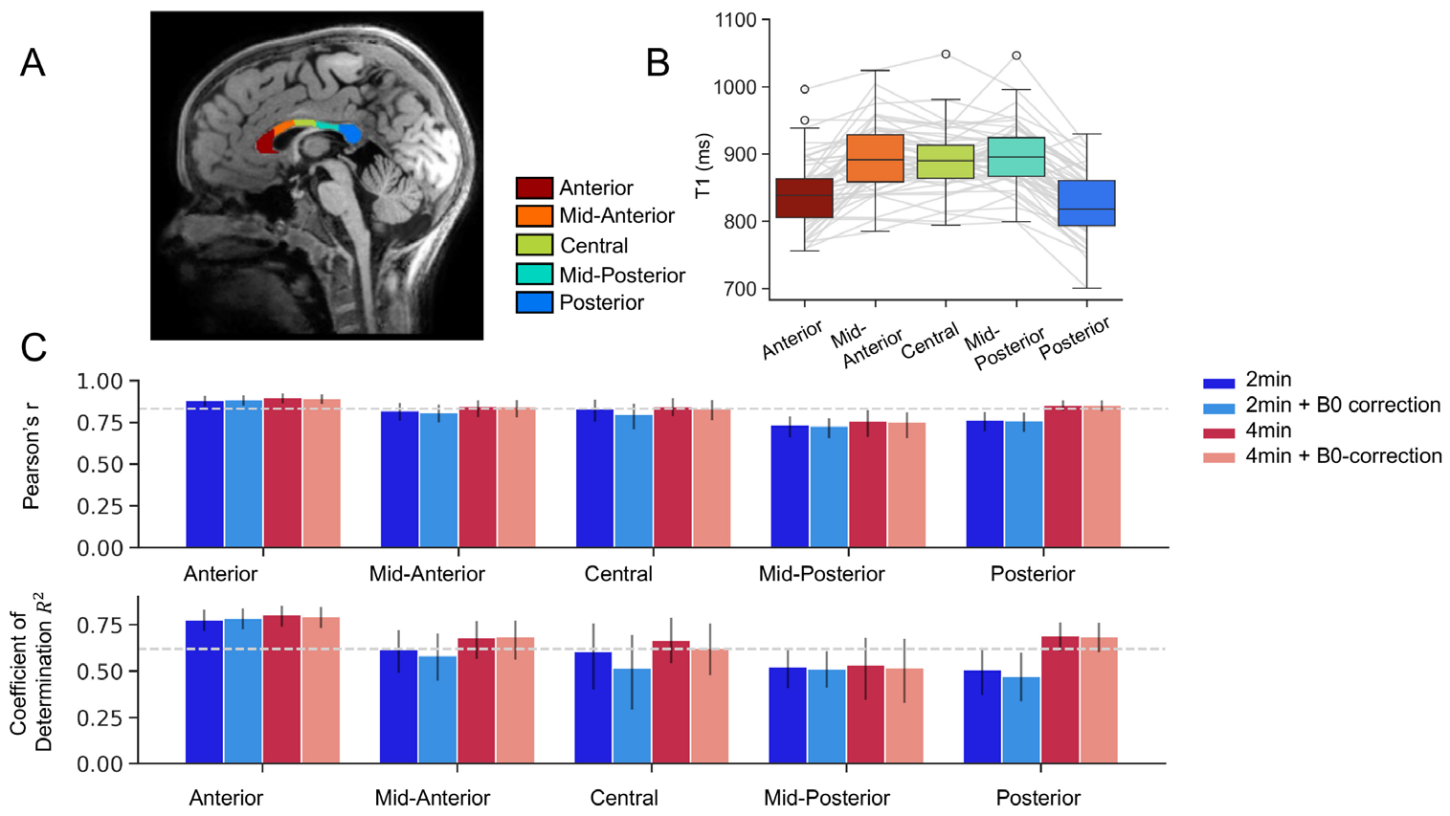
Scan–rescan reliability and T1 values in the corpus callosum (CC).(A) Five regions of interest (ROIs) of the CC overlaid on a syntheticT1w image of a representative subject. (B) T1 values exhibit an invertedU-shape along the anterior-posterior axis of the CC. Gray lines denoteindividual subjects. (C) Scan–rescan reliability in the CC ROIsis high across all pipelines, with subtle improvement for the 4-minpipelines.

We next evaluated mean T1 values in five segments of the corpus callosum (CC),obtained in each subject’s halfway space using Freesurfer. We firstobserved that T1 values follow an inverted U-shape (Fig. 5B), in line with previous findings usingtraditional quantitative T1 methods and histology (Aboitiz et al., 1992;Berman et al., 2018;Hofer et al., 2015;Lynn et al., 2021;Stikov et al., 2011,^2015^). Across the five segments, T1 values werehighly reliable for all four reconstruction pipelines, with slightly higherreliability for 4-min data (Table1;Fig. 5C).Similarly to the voxel-based analysis, B0 correction did not contribute toreliability in the CC segments.Supplementary Figure S1shows thedistribution of differences between T1 values in the two scans in the fourpipelines. Reliability of T1 values in subcortical gray matter regions was alsovery high (mean Pearson’s R > 0.9, CV < 1.5%) and isreported inSupplementary Table S1andFigure S2.

**Table 1.** Scan–rescan reliability values for the corpus callosum (CC).

	Coefficient of determination (R ^2^ )				Coefficient of variation (CV, %)			
ROI	2 min	2 min + B0	4 min	4 min + B0	2 min	2 min + B0	4 min	4 min + B0
Anterior	0.777	0.784	0.804	0.794	1.58	1.52	1.37	1.44
MidAnterior	0.616	0.584	0.680	0.684	2.02	2.04	1.81	1.80
Central	0.605	0.516	0.663	0.621	1.66	1.79	1.65	1.74
MidPosterior	0.521	0.510	0.534	0.517	2.17	2.21	1.84	1.89
Posterior	0.505	0.469	0.691	0.684	2.46	2.56	1.84	1.89

Reported are the coefficient of determination (R^2^) andcoefficient of variation (CV).

### 3.4 Reliability of T1 values in white matter tracts

We excluded high-motion diffusion scans where the mean framewise displacement(FD) was greater than 0.8 mm, or the neighborhood correlation was lower than r= 0.6. This resulted in a sample size of 41 subjects that had goodquality diffusion data in both timepoints.

**Fig. 6.**
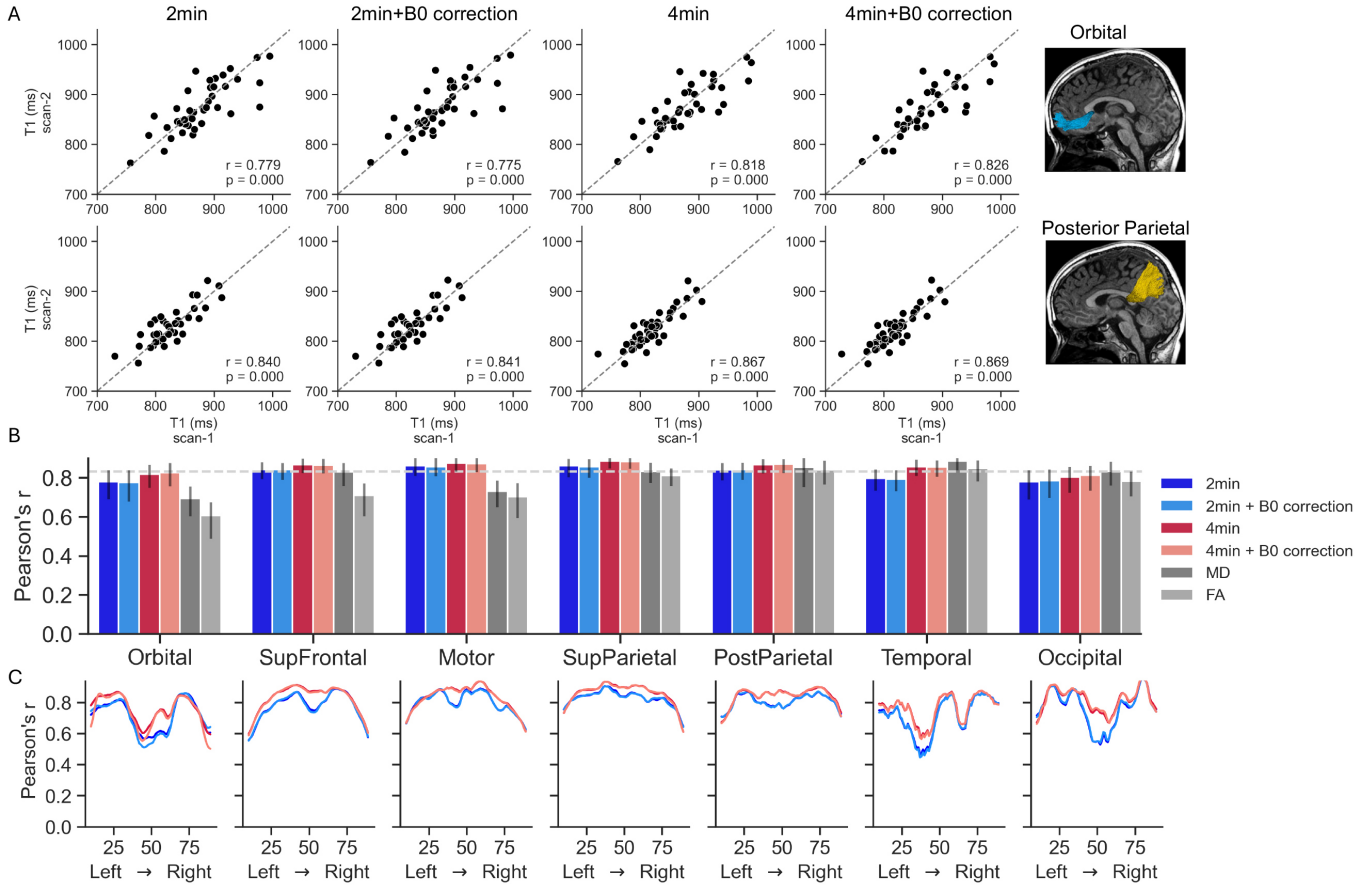
Scan–rescan reliability of callosal white matter tracts using thefour reconstruction pipelines. (A) Mean T1 values for the first andsecond scan of each participant in the orbital sub-bundle (top) andposterior parietal sub-bundle of the corpus callosum (CC). Dashed linesrepresent the equality line. (B) Pearson’s r correlationcoefficient for mean T1 values across the four pipelines for the sevenCC sub-bundles. Error bars denote the 68% confidence interval calculatedusing a bootstrap permutation procedure. Diffusion metrics are shown forreference in gray (MD, mean diffusivity; FA, fractional anisotropy).Dashed line represents the median reliability across all tracts. (C)Reliability along the tract profile. Each line denotes thePearson’s r correlation coefficient between T1 values from thetwo timepoints, across the length of the tract profile. Each linerepresents a single reconstruction pipeline. In each sub-bundle, nodesare ordered from left to right.

**Fig. 7.**
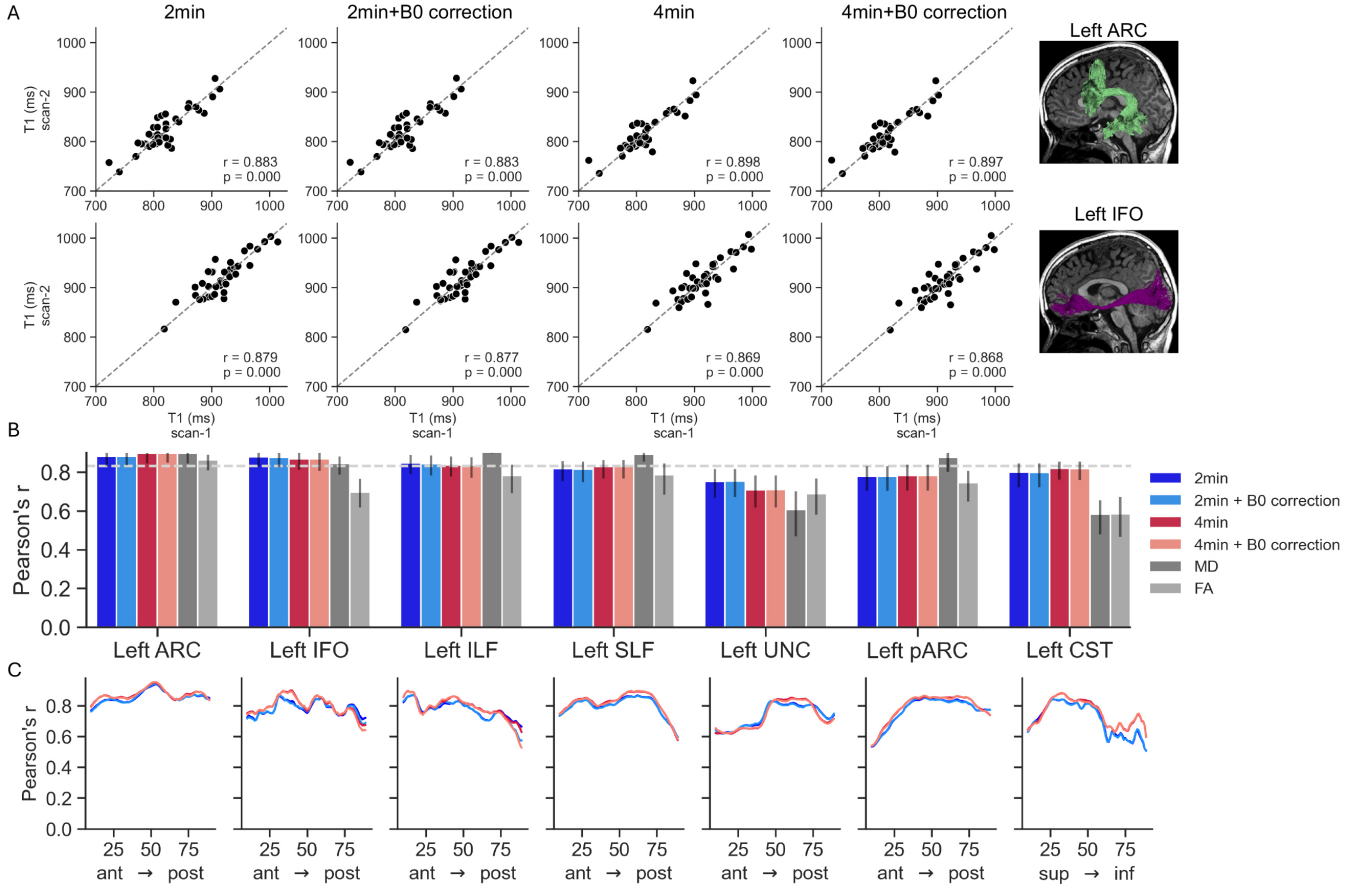
Scan–rescan reliability of left hemisphere white matter tractsusing the four reconstruction pipelines. (A) Mean T1 values for thefirst and second scan of each participant in the left arcuate fasciculus(ARC, top) and left inferior fronto-occipital fasciculus (IFO, bottom).Dashed lines represent the equality line. (B) Pearson’s rcorrelation coefficient for mean T1 values across the four pipelines inleft hemisphere tracts. Error bars denote the 68% confidence intervalcalculated using a bootstrap permutation procedure. Diffusion metricsare shown for reference in gray (MD, mean diffusivity; FA, fractionalanisotropy). Dashed line represents the median reliability across alltracts. (C) Reliability along the tract profile. Each line denotes thePearson’s r correlation coefficient between T1 values from thetwo timepoints, across the length of the tract profile. Each linerepresents a single reconstruction pipeline. In each tract, nodes areordered from anterior to posterior position.

We first evaluated scan–rescan reliability by comparing mean tract T1values across the two scans of each participant, calculated using each of thefour different pipelines. This analysis showed that across all tracts, thereliability was high even for the single 2-min scan without B0 correction (meanPearson’s r = 0.827 across 21 tracts, range [0.751–0.887]).Increasing the scan duration to 4 min slightly increased reliability (mean r= 0.836, range [0.709–0.898]), while B0 correction hardly had aneffect on reliability (mean r = 0.826, range [0.753–0.887]; mean r= 0.836, range [0.712–0.897] for 2 and 4 min, respectively). Theseresults are consistent with what we reported above for the voxel-based andROI-based analyses.Figures 6–7andSupplementary Figure S3show the variabilityin scan–rescan correlation values across different tracts, andSupplementaryTable S2reports the corresponding coefficients of determination andcoefficients of variation (CV). Across tracts, the CV remains below 3%, and iseither similar or smaller in the 4-min pipelines, suggesting that the increasedSNR yields greater stability in measurement over time.SupplementaryFigures S4–S7show the distribution of differences between T1values in the two scans in the four pipelines.

For reference, we also calculated the reliability of diffusion-based metrics foreach tract, fractional anisotropy (FA) and mean diffusivity (MD), in the samemanner. This analysis revealed that MRF-derived T1 values show comparable orhigher reliability than that of widely used diffusion-based metrics (FA: mean r= 0.754 range [0.584–0.904; MD: median r = 0.795 range[0.548–0.926]). As shown inFigures 6–7(andSupplementary Fig. S3), the improved reliability of MRF comparedwith diffusion stands out especially for tracts where diffusion metrics haverelatively low reliability, like the Uncinate Fasciculus and Corticospinaltract. This comparison does not suggest that MRF may replace diffusion data, asthey are sensitive to different tissue properties and provide complementaryinformation (Huber et al.,2021;Travis et al.,2019;Yeatman, Wandell,et al., 2014). Instead we use this comparison to highlight that thereliability of MRF is just as good or even greater than that of measurementsthat are widely used in the field.

**Fig. 8.**
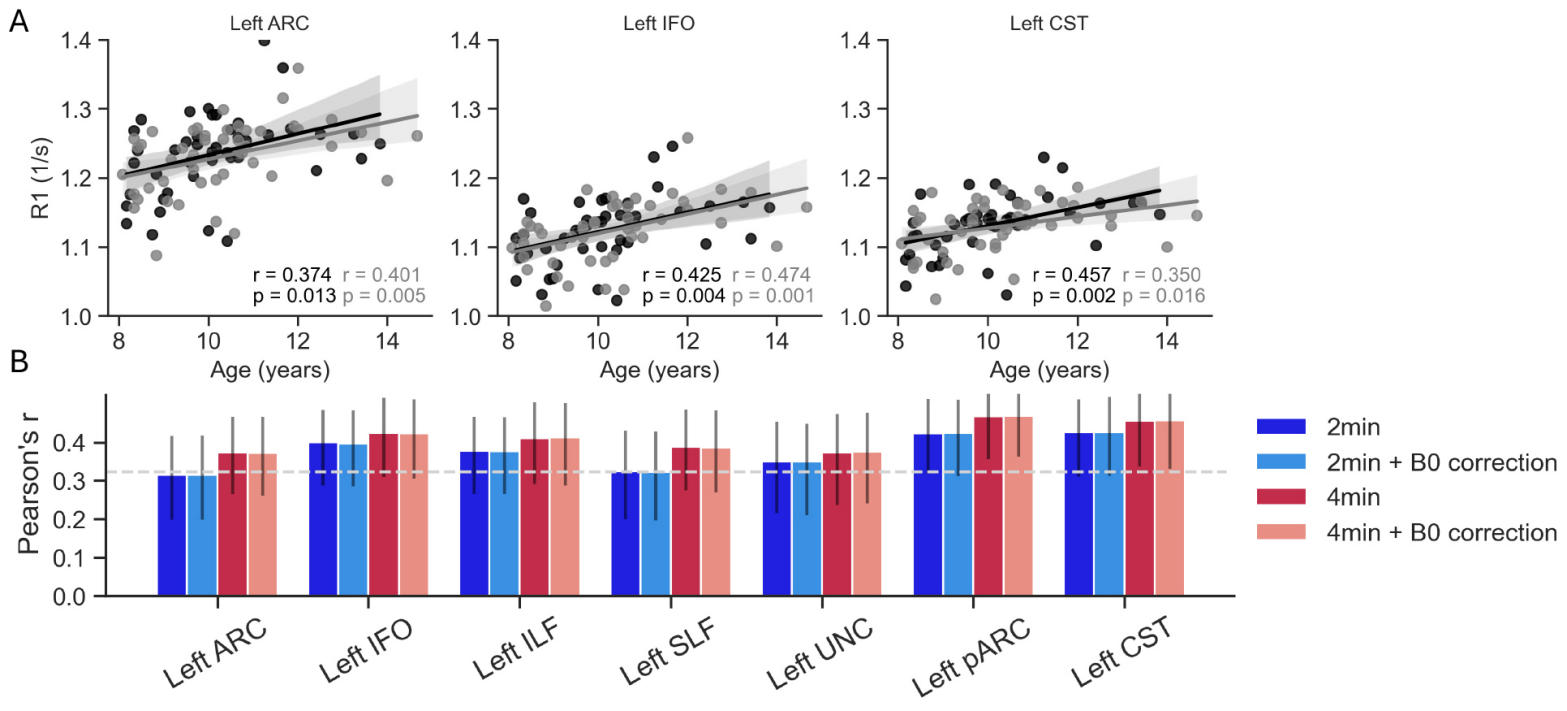
Tract mean R1 values positively correlate with age. (A) Scatterplots showR1 values calculated using the 4-min pipeline (without B0 correction)from the two scans of each participant (first scan N = 43, black;second scan N = 46, gray). Lines denote the best linear fitsurrounded by the 95% confidence interval (shaded area). Shown are theleft arcuate fasciculus (ARC), left Inferior fronto-occipital fasciculus(IFO) and left corticospinal tract (CST). (B) Correlation coefficientsbetween age and R1 values in left hemisphere tracts when calculatedusing the four different pipelines (scatterplots in A correspond to thered bars). Error bars denote the 68% confidence interval calculatedusing a bootstrap permutation procedure. The dashed line denotes theFDR-corrected significance level at p < 0.05.

Lastly, we examined whether R1 (1/T1) values along different tracts replicateknown age effects. We found that R1 was positively correlated with age inmultiple intra-hemispheric tracts, as well as in the more frontal sub-bundles ofthe corpus callosum (Fig. 8;Supplementary Fig. S8), in line with previous findings (Yeatman, Wandell, et al.,2014). Repeating this analysis using the four pipelines revealed similarresults. Interestingly, there was a trend where in some tracts age effects werelarger when R1 values were calculated with 4-min pipelines, compared with the2-min pipelines. While not statistically significant, this might suggest thatusing 4-min sequences increases SNR and enhances the ability to captureunderlying effects of tissue development.

## 4 Discussion

In this study, we evaluated the reliability of a novel and rapid MRF sequence thatcan be used to obtain high-quality quantitative T1 data in young children. Ouranalyses showed that MRF-derived T1 values were highly reliable in white matter overtime, and were able to capture known age effects. In addition, we evaluated thecontribution of different acquisition and preprocessing choices onscan–rescan reliability.

Our analyses revealed that high scan–rescan reliability in white matter can beachieved with a single 2-min scan and that reliability increases with scan. Whileprevious studies evaluated the reliability of MRF in phantoms and healthy adults(Buonincontri et al., 2019;Gracien et al., 2020;Körzdörfer et al.,2019;Wicaksono et al.,2023), here we assessed the scan–rescan reliability in a real liferesearch scenario with young children. Our results provide a proof of concept forthe feasibility of using MRF to measure tissue properties reliably over time withpopulations where data acquisition and quality are more challenging. These resultshold important implications for the field of developmental cognitive neuroscienceand pave the way for studies that focus on structural brain development inchildren.

We examined the reliability of MRF-derived T1 values in white matter using threecomplementary methodologies, namely, comparing individual voxels, analyzing specificROIs, and using diffusion-based tractography. This three-pronged approach wasintended to test MRF-derived values in the common analyses used to study whitematter structure. Together our results show high scan–rescan reliability ofMRF in white matter, independent of the downstream analysis method. The voxel-wiseanalysis allowed us to describe variation in reliability across individuals. The ROIapproach was useful to capture the canonical U-shape of the corpus callosum andcompare it with existing literature. Lastly, diffusion tractography has beenextensively used to study the relationship between white matter development andcognitive functions (Roy et al.,2024;Wang et al.,2017;Yeatman, Dougherty,Ben-Shachar, et al., 2012). We show that using a standard automatedpipeline for tractography, the reliability of MRF-derived tract profiles isremarkably high across the tracts examined, and in some cases exceeded thereliability of diffusion-based metrics.

In addition to establishing the reliability of the MRF sequence, the current workvalidates MRF-derived T1 values in two ways. We first found that T1 values in thecorpus callosum follow an inverted U-shape, in line with prior observations usingother quantitative methods and histology (Aboitiz et al., 1992;Berman et al., 2018;Hofer et al., 2015;Lynn et al., 2021;Stikov et al., 2011,^2015^). This demonstrates that MRF-derived T1 values aresensitive enough to capture this variation in adjacent parts of the corpus callosum.In addition, we observed significant correlations between age and R1 values inmultiple intra-hemispheric white matter tracts, replicating known age effects (Callaghan et al., 2014;Eminian et al., 2018;Yeatman, Wandell, et al., 2014).Interestingly, the only callosal sub-bundles that correlated with age were theanterior ones, in line withYeatman,Wandell, et al. (2014). The observed age effects may reflect severaldevelopmental processes, since R1 has been shown to be sensitive to water contentand iron composition (Fatouros et al.,1991;Filo et al.,2023;Gelman et al.,2001), in addition to its well-established relationship with myelincontent (Lutti et al., 2014;Stüber et al., 2014).Importantly, these processes are not mutually exclusive through development, asincrease in myelin content is tightly linked to decrease in water content in thesame regions and may be accompanied by local changes in the molecular composition ofthe myelin sheath. One interpretation of the observed increase in R1 throughdevelopment is as reflecting the prolonged myelination of white matter which lastsinto adulthood. In our age range, we were able to capture linear growth in R1values, yet studies with a wider age range will be needed to determine whether therelationship between age and R1 remains linear or plateaus later in adolescence.Together, this shows that MRF-derived T1 values capture similar effects to standardT1 values acquired with longer acquisitions. The short duration of the MRF sequenceand its ability to capture multiple quantitative maps in a single acquisition makeit feasible for future studies to combine several indices to disentangle the tissueprocesses underlying neurodevelopment, disease, and aging.

One of the principal strengths of this work lies in its implementation within acohort of children scanned multiple times, which offers promise for quantitative MRIadoption in brain development research. A central challenge in imaging youngchildren is motion during scans, which significantly compromises data quality andleads to high rates of data exclusion (Afacan et al., 2016;Nebelet al., 2022;Satterthwaiteet al., 2012). Importantly, data exclusion based on motion may lead tobiased samples that underrepresent younger children or those that suffer from moreextreme phenotypes (Nebel et al.,2022;Wylie et al.,2014). The proposed MRF protocol overcomes this barrier by employing afast and robust sequence with spiral readout, which is less likely to be affected bymotion (Glover & Pauly,1992). Using short sequences not only mitigates motion artifacts inacquired data, but also increases the probability of acquiring the datasuccessfully, as it is not uncommon for children to end scanning sessionsprematurely before completing the full sequence. With a protocol that takes2–4 min, this risk is greatly reduced compared with standard protocols thattake about 20 min to complete and require a sequence of images. This may also reducethe drop-out rate in follow-up visits which is a major challenge in longitudinalstudies. Based on the pipeline comparison we conducted, below we provide a list ofpractical recommendations for researchers and clinicians interested in using MRFsequences in research and practice.

### 4.1 Practical recommendations

#### 4.1.1 Scan duration

Our findings show that reliability increased when we combined two 2-minsequences compared with a single run. Importantly, even for the single 2-minsequence, scan–rescan reliability was greater than r = 0.7,and that for white matter tracts was comparable or better than thescan–rescan reliability of diffusion metrics. We recommend that iftime allows, two repetitions will boost SNR and reliability, but forclinical studies and situations where time is a limiting factor, a single2-min sequence could provide usable quantitative T1 data for manyapplications.

#### 4.1.2 B0 correction

Our findings do not indicate a significant improvement in reliability ofwhite matter T1 values by including a separate B0 correction. B0 correctionwill likely be more important for studies that target orbital regions in thevicinity of the sinuses, which may suffer more from inhomogeneity (seeFig. 2). B0 correction mayalso prove crucial for accurate T2 maps, but this remains to be assessed infuture work.

#### 4.1.3 Registration

In order to accurately evaluate the reliability of MRF-derived T1, we createda half-way space template image and simultaneously resampled both scansessions into the half-way space (Reuter & Fischl, 2011;Reuter et al., 2010). Thecommon practice of registering one scan to another creates an inherentasymmetry in the sense that the second scan is aligned, registered, andresampled to match the spatial configuration of the first scan, while theinitial scans remain unaltered. The disparity introduces a systematic biasinto the repeatability analysis, such that the values may differ dependingon which scan serves as the reference image and which scan is undergoing theregistration and resampling. We propose that registration to a half-wayspace resolves this asymmetry and leads to more accurate results.

### 4.2 Limitations and future directions

The current protocol and preprocessing pipeline do not provide information aboutmotion during the scan. The evaluation of motion was done qualitatively based onthe reconstructed images. Quantifying motion would allow us to explicitlyquantify the effect of motion on MRF-derived T1 values, and potentially explainsome of the variance in scan–rescan reliability across subjects. Anotherlimitation of the current protocol is that it did not include B1 inhomogeneitymapping, which is an essential step for generating accurate T2 maps from the MRFsequence. Future versions of the MRF sequence may incorporate B1 quantificationto fully exploit MRF capabilities to obtain multiple quantitative mapssimultaneously (e.g., T2, proton density). Lastly, a technical challenge forimplementing this protocol widely in clinical settings is that thereconstruction phase is currently lengthy and requires heavy computations.Future work can improve the efficiency of the reconstruction making it feasiblein clinical settings (Zhou et al.,2024) while also including online motion correction (Nurdinova et al., 2024).

## 5 Conclusion

In sum, MRF provides a promising methodology for deriving reliable quantitativemetrics of brain tissue structure in children and patient populations where scantime and motion are of particular concern. Coupled with the pipeline we proposewhich improves the repeatability, the current work paves the way for using MRF inlongitudinal pediatric studies. This method would facilitate studies focusing onstructural brain development by providing an easy and rapid way to quantify changesin myelin and other properties of white matter.

## Supplementary Material

Supplementary Material

## Data Availability

Data associated with this publication is publicly available here:https://purl.stanford.edu/gz224th5011
