# Supplementary material for: Fast and reliable quantitative measures of white matter development with magnetic resonance fingerprinting
Source: Imaging Neurosci (Camb). 2025 Feb 18;3:imag_a_00470. doi: 10.1162/imag_a_00470 (PMC12319766; doi:10.1162/imag_a_00470)
Supplement: Supplementary Material [file imag_a_00470-supp.pdf]

Fast and reliable quantitative measures of white matter development with magnetic resonance fingerprinting

Maya Yablonski*[1,2], Zihan Zhou*[2,3], Xiaozhi Cao[3,4], Sophie Schauman[3], Congyu Liao[3,4], Kawin Setsompop[3,4], Jason D. Yeatman[1,2,5]

[1] Division of Developmental-Behavioral Pediatrics, Department of Pediatrics, Stanford School of Medicine
[2] Graduate School of Education, Stanford University
[3] Department of Radiology, Stanford University
[4] Department of Electrical Engineering, Stanford University
[5] Department of Psychology, Stanford University

***equal contribution**

Supplementary material

	Coefficient of Determination (R^2)				Coefficient of Variation (CV, %)			
**ROI**	**2min**	**2min + B0**	**4min**	**4min + B0**	**2min**	**2min + B0**	**4min**	**4min + B0**
Left Caudate	0.843	0.846	0.917	0.874	1.39	1.31	1.06	1.08
Left Putamen	0.883	0.883	0.899	0.899	1.32	1.33	1.10	1.11
Left Thalamus	0.664	0.696	0.826	0.850	1.58	1.55	1.13	1.08
Right Caudate	0.890	0.893	0.906	0.906	1.30	1.29	1.23	1.21
Right Putamen	0.871	0.870	0.889	0.890	1.40	1.41	1.13	1.12
Right Thalamus	0.807	0.803	0.886	0.890	1.37	1.37	1.03	1.03

Supplementary Table S1. Coefficient of determination (R2) and Coefficient of variation (CV%) comparing mean T1 values in 6 subcortical gray matter regions, across two timepoints using each of the four pipelines.

	Coefficient of Determination (R^2)				Coefficient of Variation (CV, %)			
**Tract**	**2min**	**2min + B0**	**4min**	**4min + B0**	**2min**	**2min + B0**	**4min**	**4min + B0**
ARC_L	0.765	0.765	0.799	0.797	1.30	1.30	1.11	1.11
IFO_L	0.757	0.755	0.738	0.738	1.15	1.15	1.18	1.17
ILF_L	0.673	0.665	0.657	0.651	1.59	1.60	1.65	1.65
SLF_L	0.623	0.619	0.664	0.664	1.85	1.86	1.64	1.65
UNC_L	0.529	0.531	0.462	0.465	2.30	2.30	2.52	2.51
pARC_L	0.553	0.550	0.569	0.570	1.86	1.85	1.66	1.66
CST_L	0.609	0.603	0.655	0.650	1.39	1.40	1.20	1.21
ARC_R	0.766	0.766	0.781	0.780	1.31	1.30	1.21	1.21
IFO_R	0.736	0.740	0.690	0.692	1.21	1.20	1.23	1.23
ILF_R	0.737	0.736	0.676	0.674	1.40	1.41	1.48	1.49
SLF_R	0.683	0.684	0.664	0.666	1.56	1.55	1.56	1.55
UNC_R	0.491	0.490	0.521	0.529	1.85	1.85	1.89	1.85
pARC_R	0.680	0.681	0.644	0.641	1.65	1.64	1.62	1.63
CST_R	0.603	0.600	0.670	0.669	1.38	1.37	1.14	1.15
Orbital	0.573	0.565	0.659	0.663	1.96	1.93	1.82	1.77
SupFrontal	0.692	0.691	0.744	0.740	1.64	1.65	1.45	1.47
Motor	0.731	0.727	0.756	0.752	1.71	1.75	1.59	1.63
SupParietal	0.719	0.713	0.767	0.765	1.43	1.46	1.29	1.30
PostParietal	0.674	0.675	0.721	0.723	1.46	1.47	1.27	1.26
Temporal	0.625	0.619	0.733	0.729	2.75	2.78	2.24	2.26
Occipital	0.498	0.512	0.587	0.606	1.55	1.55	1.47	1.43

Supplementary Table S2. Coefficient of determination (R2) and Coefficient of variation (CV%) comparing mean T1 values in 21 white matter tracts, across two timepoints using each of the four pipelines.

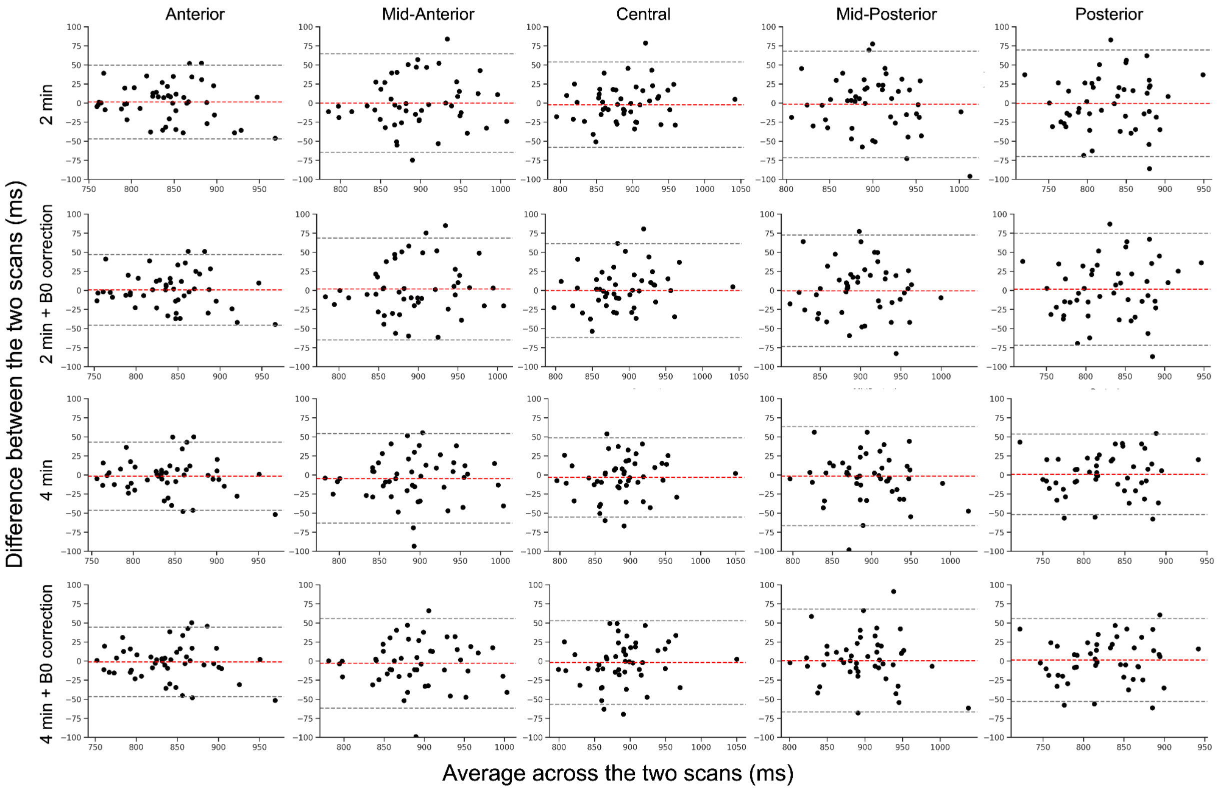

Figure S1. Bland-Altman plots of the T1 values in corpus callosum regions, as calculated with different reconstruction pipelines. In each plot, the red line denotes the mean difference between the two scans. The dashed gray lines denote the limits of agreement, defined as the mean difference ±1.96 *standard deviation of the difference (equivalent to 95% confidence interval).

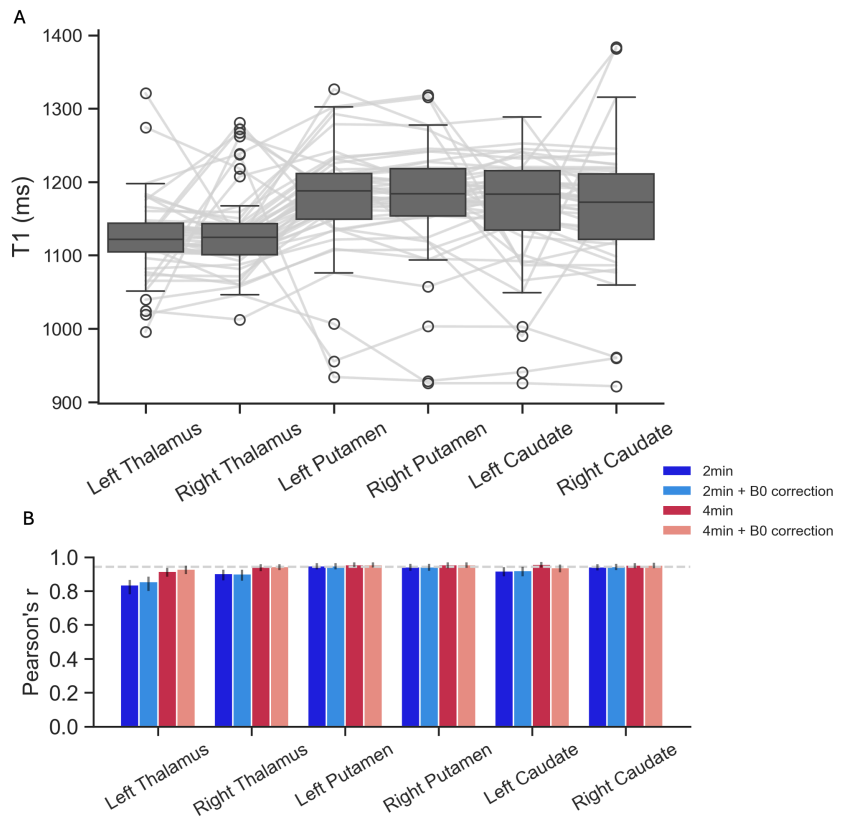

Figure S2. Scan-rescan reliability of T1 values in subcortical regions. A) Distribution of T1 values extracted from 6 gray matter subcortical regions using the 4-minute pipeline without B0 correction. B) Test-retest reliability across the two timepoints calculated with each reconstruction pipeline. Error bars denote the 68% confidence interval calculated using a bootstrap permutation procedure with 10000 iterations. Dashed line represents the median reliability across all subcortical regions.

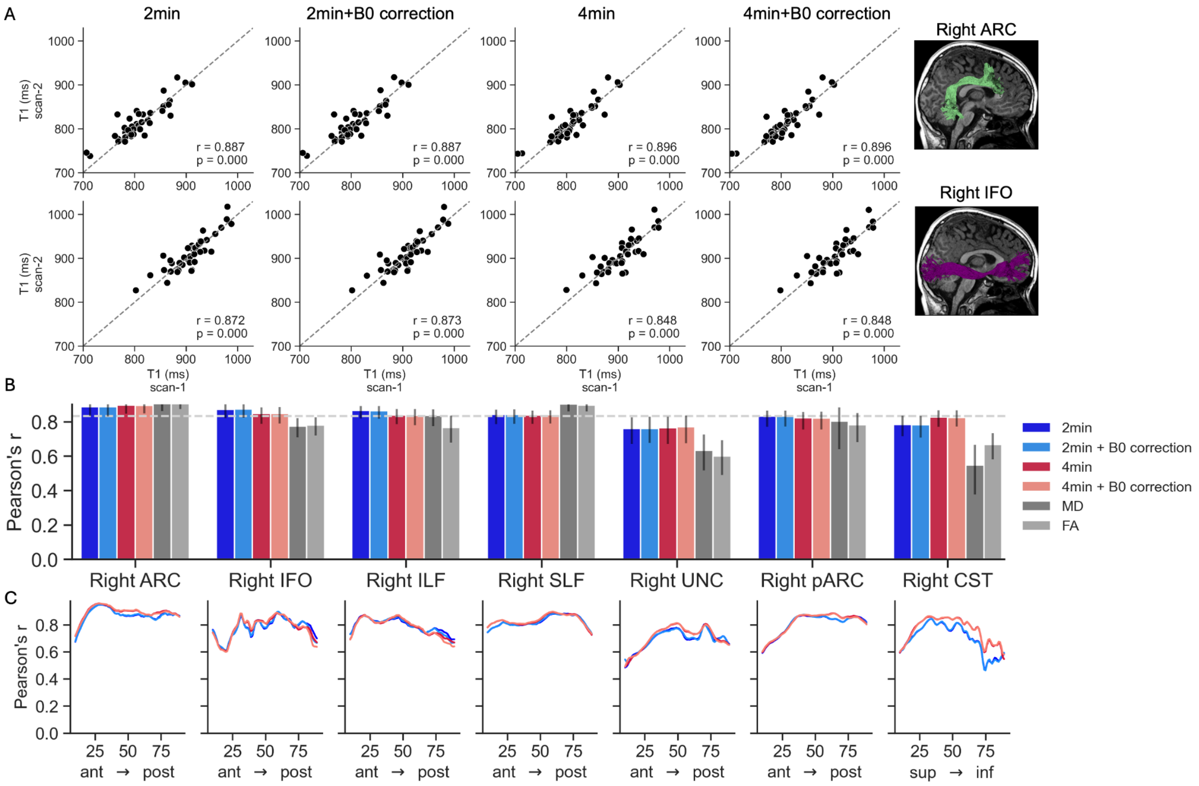

Figure S3. Scan-rescan reliability of right hemisphere white matter tracts using the 4 reconstruction pipelines. A) mean T1 values for the first and second scan of each participant in the right arcuate fasciculus (ARC, top) and right inferior fronto-occipital fasciculus (IFO, bottom). Dashed lines represent the equality line. B) Pearson's R correlation coefficient for mean T1 values across the 4 pipelines in right hemisphere tracts. Diffusion metrics are shown for reference in gray (MD, mean diffusivity; FA, fractional anisotropy). Dashed line represents the median reliability across all tracts. Error bars denote the 68% confidence interval calculated using a bootstrap permutation procedure with 10000 iterations. C) Reliability along the tract profile. In each tract, nodes are ordered from anterior to posterior position.

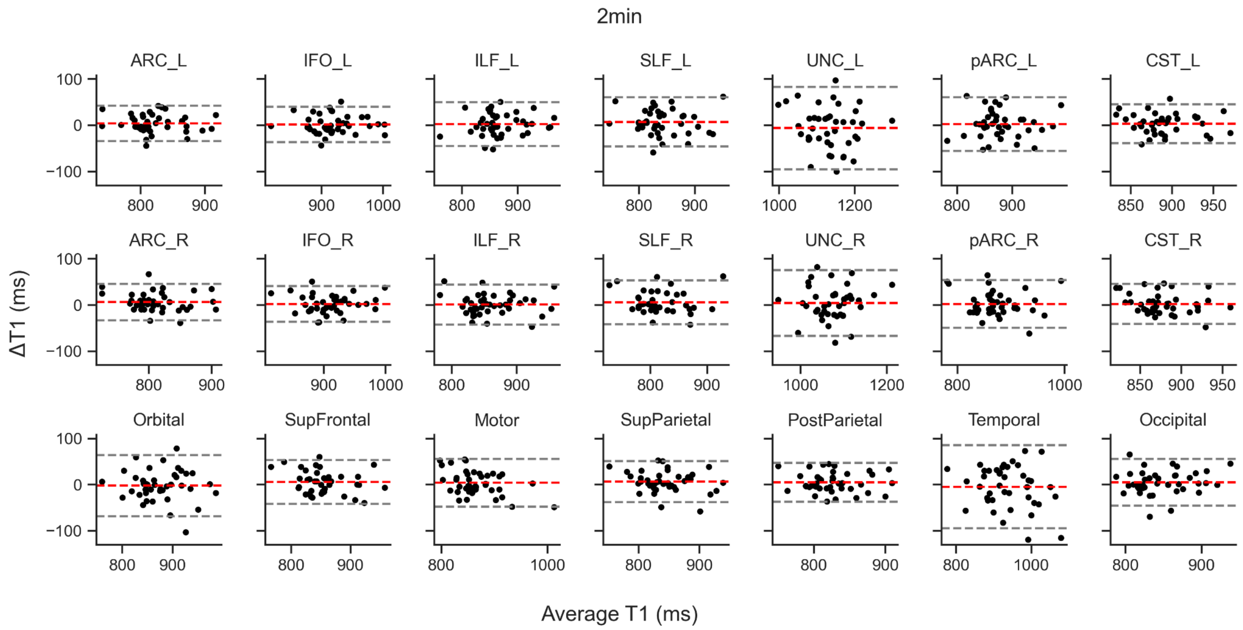

Figure S4. Bland-Altman plots of the mean T1 values in the 21 white matter tracts, calculated with the two minute pipeline. In each plot, the red line denotes the mean difference between the two scans. The dashed gray lines denote the limits of agreement, defined as the mean difference ±1.96 *standard deviation of the difference (equivalent to 95% confidence interval). Note that the range of the x-axis varies by tract to account for the differences in mean T1 values for different tracts, while the y-axis (the difference between scans) remains constant.

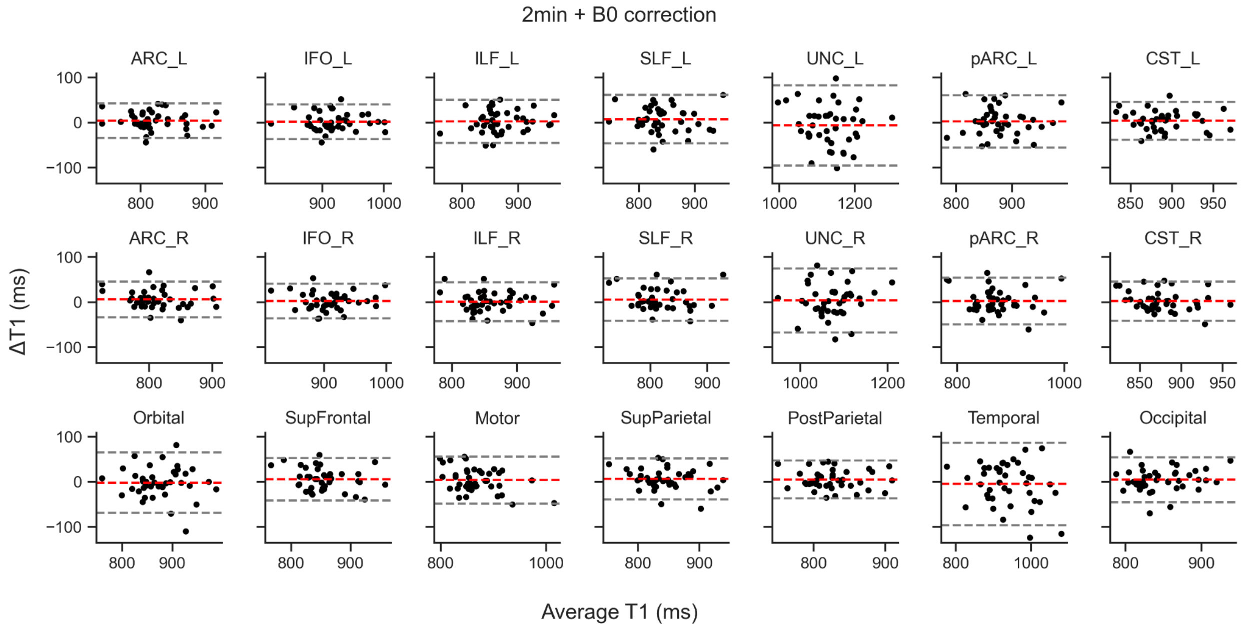

Figure S5. Bland-Altman plots of the mean T1 values in the 21 white matter tracts, calculated with the two minute + B0 correction pipeline. In each plot, the red line denotes the mean difference between the two scans. The dashed gray lines denote the limits of agreement, defined as the mean difference ±1.96 *standard deviation of the difference (equivalent to 95% confidence interval). Note that the range of the x-axis varies by tract to account for the differences in mean T1 values for different tracts, while the y-axis (the difference between scans) remains constant.

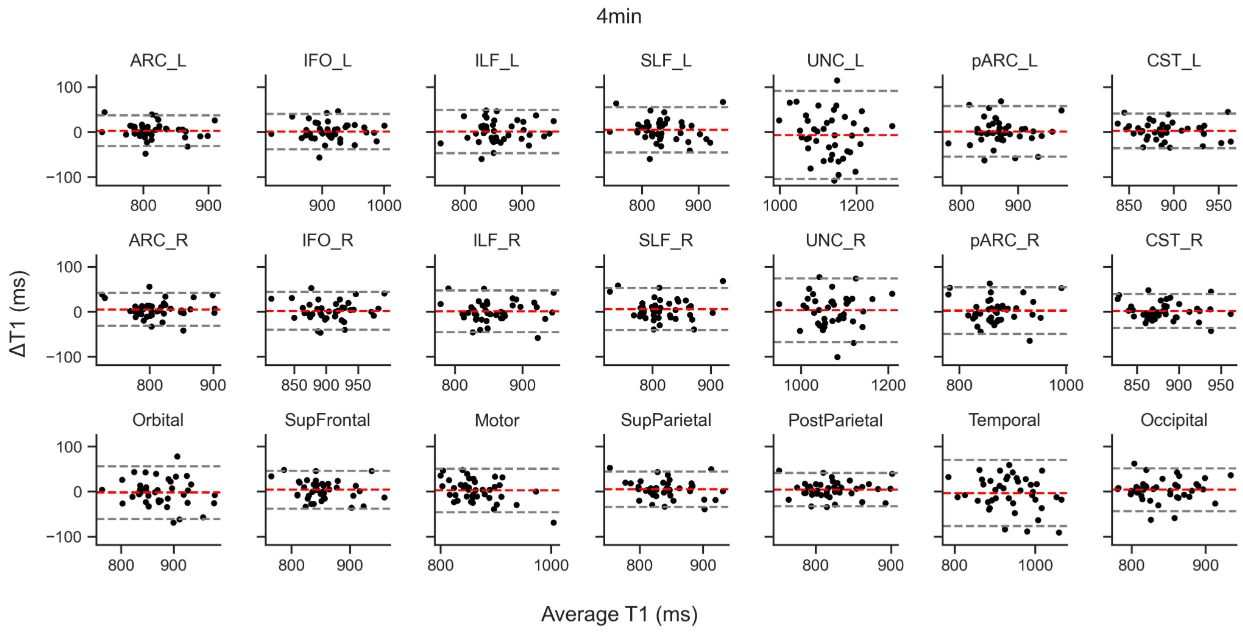

Figure S6. Bland-Altman plots of the mean T1 values in the 21 white matter tracts, calculated with the four minute pipeline. In each plot, the red line denotes the mean difference between the two scans. The dashed gray lines denote the limits of agreement, defined as the mean difference ±1.96 *standard deviation of the difference (equivalent to 95% confidence interval). Note that the range of the x-axis varies by tract to account for the differences in mean T1 values for different tracts, while the y-axis (the difference between scans) remains constant.

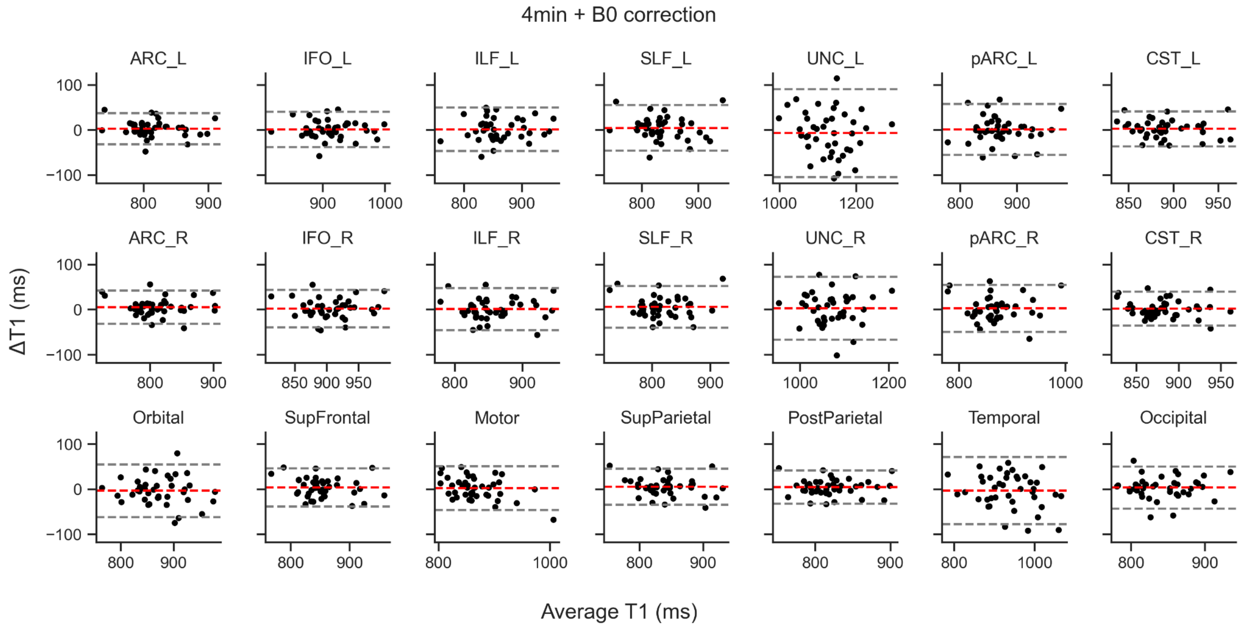

Figure S7. Bland-Altman plots of the mean T1 values in the 21 white matter tracts, calculated with the four minute pipeline with B0 correction. In each plot, the red line denotes the mean difference between the two scans. The dashed gray lines denote the limits of agreement, defined as the mean difference ±1.96 *standard deviation of the difference (equivalent to 95% confidence interval). Note that the range of the x-axis varies by tract to account for the differences in mean T1 values for different tracts, while the y-axis (the difference between scans) remains constant.

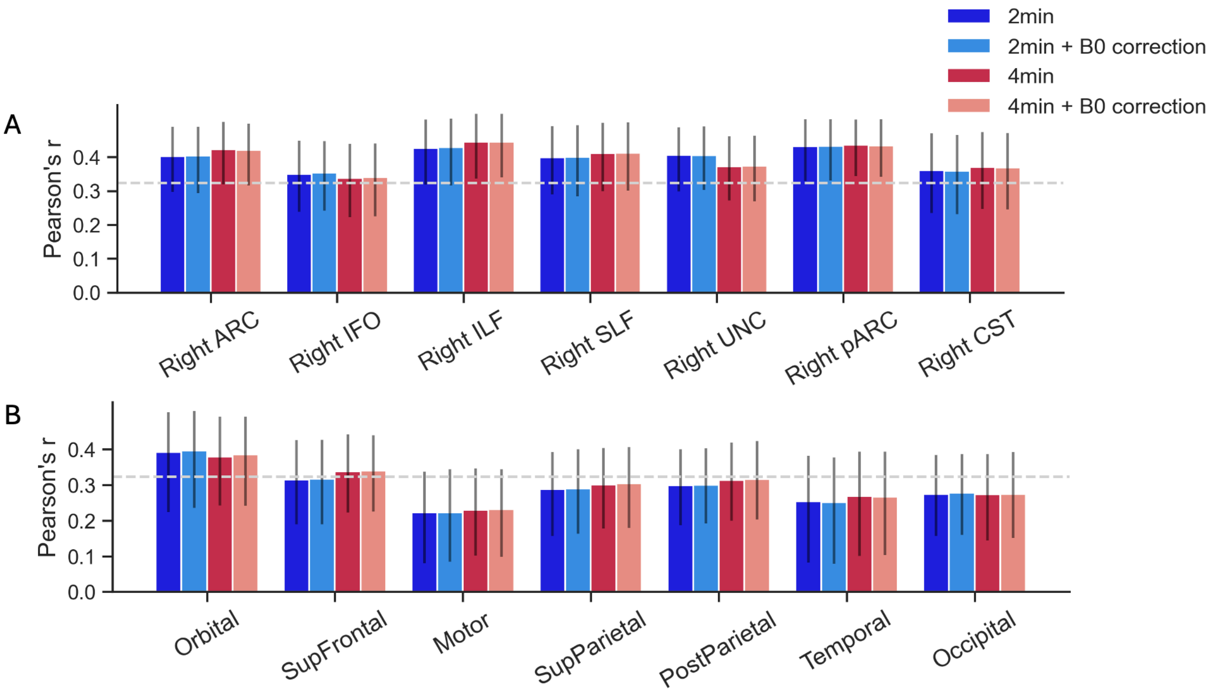

Figure S8. Correlation coefficients between age and mean R1 values calculated using the four different pipelines, in right hemisphere tracts (A) and callosal sub-bundles (B). This figure parallels Figure 8B in the main text. Error bars denote the 68% confidence interval calculated using a bootstrap permutation procedure with 10000 iterations. The dashed line denotes the FDR corrected significance level at p<0.05.
